# Effect of Salt Stress on Mutation and Genetic Architecture for Fitness Components in *Saccharomyces cerevisiae*

**DOI:** 10.1534/g3.120.401593

**Published:** 2020-08-25

**Authors:** Christopher Kozela, Mark O. Johnston

**Affiliations:** Department of Biology, Dalhousie University, Halifax, Nova Scotia Canada B3H 4R2

**Keywords:** canalization, distribution of effects of mutations, environmental variance, genetic variance, mutation, mutation rate, mutational variance, mutational heritability, *Saccharomyces*, stress, yeast

## Abstract

Mutations shape genetic architecture and thus influence the evolvability, adaptation and diversification of populations. Mutations may have different and even opposite effects on separate fitness components, and their rate of origin, distribution of effects and variance-covariance structure may depend on environmental quality. We performed an approximately 1,500-generation mutation-accumulation (MA) study in diploids of the yeast *Saccharomyces cerevisiae* in stressful (high-salt) and normal environments (50 lines each) to investigate the rate of input of mutational variation (*V*_m_) as well as the mutation rate and distribution of effects on diploid and haploid fitness components, assayed in the normal environment. All four fitness components in both MA treatments exhibited statistically significant mutational variance and mutational heritability. Compared to normal-MA, salt stress increased the mutational variance in growth rate by more than sevenfold in haploids derived from the MA lines. This increase was not detected in diploid growth rate, suggesting masking of mutations in the heterozygous state. The genetic architecture arising from mutation (M-matrix) differed between normal and salt conditions. Salt stress also increased environmental variance in three fitness components, consistent with a reduction in canalization. Maximum-likelihood analysis indicated that stress increased the genomic mutation rate by approximately twofold for maximal growth rate and sporulation rate in diploids and for viability in haploids, and by tenfold for maximal growth rate in haploids, but large confidence intervals precluded distinguishing these values between MA environments. We discuss correlations between fitness components in diploids and haploids and compare the correlations between the two MA environmental treatments.

The rate at which new mutations appear and the distribution of their effects on fitness are fundamentally important properties that can influence a variety of biological phenomena including the evolution of sex, recombination, rate of self-fertilization, diploidy, mate choice and Y-chromosome degeneration (Charlesworth and Charlesworth 1998, Keightley and Otto 2006); rate of adaptation (Covert *et al.* 2013); levels of standing genetic variation (Charlesworth and Hughes 2000) and risk of population extinction (Lande 1995, Lynch *et al.* 1995). Mutations also influence the genetic variance-covariance structure of phenotypic traits and fitness components, and thus affect evolvability (Hansen 2006), adaptation and diversification of populations. Mutations might furthermore enhance susceptibility to environmental variation, causing a decrease in canalization (Hansen 2006). Experimental studies of mutation rates and effects have been investigated largely in laboratory populations of model organisms (see Baer *et al.* 2007, Halligan and Keightley 2009 for reviews) under non-challenging conditions. Organisms under natural conditions, however, experience a range of environmental qualities, ranging from salubrious to stressful to lethal, and these environments might differ in both the propensity to induce mutation and the distribution of phenotypic effects. Stressful environments in particular may elevate the mutation rate by being directly mutagenic, by changing the chemical environment within the organism or by reducing the efficiency of repair. Although the study of mutation induction by radiation and chemicals has a rich history beginning with Muller and Stadler (see Crow and Abrahamson 1997), we have much less understanding of how environments typically experienced by organisms in nature affect the rate of mutation.

Mutations with phenotypic effects are expected to arise at different rates corresponding to the number of base pairs, or target size, influencing the trait (*e.g.*, Keightley and Ohnishi 1998, Concepcion *et al.* 2004, Landry *et al.* 2007, Lang and Murray 2008, Watanabe *et al.* 2013). Fitness itself may have the largest target size, presumably followed by fitness components such as lifespan, number of reproductive bouts and number of offspring per bout. Large target size combined with pleiotropy furthermore suggests that mutations will often affect multiple fitness components, influencing their rate of evolution (Walsh and Blows 2009). In the yeast *Saccharomyces cerevisiae*, for example, Hill and Otto (2007) found a positive correlation between mutational effects on measures of sexual and asexual fitness.

A common approach to study mutations with phenotypic effects involves the parallel propagation of a number of independent evolutionary lines derived from a single genotype that evolve under conditions where selection is less effective (Eyre-Walker and Keightley 2007). Inferences of mutation properties can be made by examining the resulting lines after a period of mutation accumulation (MA) and by comparison to the ancestral, mutation-free condition. The yeast *Saccharomyces cerevisiae* represents one of the best-studied model organisms, both generally in terms of its genetics and cell biology as well as specifically in the range of MA studies where it has been used. Zeyl and DeVisser (2001) first measured genome-wide mutation rates affecting fitness in diploid yeast. Their fitness measure, asexual mitotic growth rate, was chosen because of its strong association with total fitness along with its property of being a large mutational target. Subsequent studies have investigated genome-wide mutation for a range of other characters, including haploid growth (Wloch *et al.* 2001, Hall and Joseph 2010), haploid viability (Hall and Joseph 2010) and sporulation (Hill and Otto 2007, Hall and Joseph 2010). Like many of the historical studies of mutation, these recent studies have left us with perhaps as many questions as answers regarding basic mutation parameters in evolving populations. Mutation rates affecting growth rate seem to be low, but estimates vary by nearly an order of magnitude (Zeyl and DeVisser 2001, Hill and Otto 2007). Rates affecting sporulation were found to be much larger than those affecting growth rate (Hill and Otto 2007, Hall *et al.* 2008, Hall and Joseph 2010), with a corresponding decrease in the average size of mutational effect. Other mutation parameters have been estimated in these studies, such as those describing the overall distribution of effects as well as the proportion of mutations that are beneficial (see also Dickinson 2008) but with little consensus.

In this study we ask how a suboptimal — that is, stressful — environment influences mutational variance, the mutation rate and distribution of effects. We propagated a series of MA lines where one-half of the lines were cultured in a high sodium chloride (NaCl) environment, while the other half were propagated in a standard yeast culture environment. High NaCl has numerous effects on *S. cerevisiae* and is one of the best-studied stressful environments. It broadly fits into two classes of stress, osmotic stress and ionic stress. Osmotic stress in yeast activates the high-osmolarity glycerol (HOG) signaling pathway (Hohmann 2008), causing an increase in the concentration of intracellular glycerol and mitigating the effects of high extracellular osmolyte. Additional downstream events in this pathway affect numerous transcriptional responses including the transient cessation of the cell cycle and protein synthesis. Ionic stress results in the activation of additional ion transport mechanisms that act to restore balance across the cell membrane. It has been shown that NaCl can induce oxidative stress and DNA damage in cultured mammalian cells (Kültz and Chakravarty 2001, Zhang *et al.* 2004) as well as sensitizing cells to other mutagenic treatments (Han *et al.* 2007, Zhu *et al.* 2008). DNA damage responses are also involved in the generalized yeast stress response (Gasch and Werner-Washburne 2002), a program of transcriptional responses induced by a wide range of stressful environments.

Using the MA approach, we propagated 50 lines in each environment for approximately 1,500 cell generations. Lines were then assayed for fitness traits in standard culture conditions. Specifically, we estimated mutation parameters for diploid growth rate (a measure of asexual fitness) and sporulation efficiency (a measure of sexual fitness). We then derived haploids from a sample of the MA lines and measured haploid viability and growth rate. Mutation parameters were thus estimated for four fitness components in lines generated in two MA environments.

## Materials and Methods

### Mutation accumulation

We used a diploid isolate of *Saccharomyces cerevisiae* PP271.1, a W303-like strain, to inoculate a batch culture in 200 ml YPD (yeast extract peptone dextrose). This culture was grown for 48 hr at 28° while shaking and constituted the ancestral population. Replicate 1.5 ml portions of this culture were frozen at -80° in 20% glycerol. This population was used to initiate a series of 50 mutation accumulation (MA) lines cultured on solid YPD media (normal-MA treatment) and 50 MA lines cultured on YPD made with 1.0M NaCl (salt-MA treatment), a level of salt that slows the growth of this strain by approximately 50% at 28°. The 100 lines were routinely bottlenecked to a single individual by streaking a haphazardly chosen colony using a sterile swab onto a fresh media plate. Prior to transfer, colonies were photographed on a stereo dissection microscope and images were used later in the estimation of generation number from colony area based on the relation between colony area and cell number (see Supplemental Material File S1). All cultures were grown at 28° in a growth cabinet with plate position shuffled randomly at each transfer. Lines cultured on normal medium were transferred to fresh plates (bottlenecked) daily for 75 days. We wished to equalize the number of generations between treatments; therefore, lines cultured on salt were transferred every 2 days to fresh medium for 81 transfers over 162 days. To ensure that treatments differed only in salt concentration, we prepared media for both treatments as a single batch prior to the addition of NaCl to the salt medium. During the early course of MA, we observed a marked decline in the growth rates of colonies on the salt medium. At transfer 26 we therefore decreased the concentration of NaCl to 0.9M for the remainder of the MA phase. During the course of MA, two lines of the normal treatment and three lines of the salt treatment went extinct. Using the information from the colony sizes at each transfer, we estimated the average effective population size (*N*_e_) by taking the harmonic mean for lines in each treatment. On the assumption that each transfer starts from a single cell and that the population doubles until the average population size at transfer is reached, the *N*_e_ of both the normal and salt treatments were found to be approximately 10.5 over the course of MA.

Upon completion of approximately 1,503 (normal-MA) and 1,573 (salt-MA) generations, lines were expanded into late-log-phase populations by inoculating 5ml liquid culture of YPD and salt YPD with lines from the appropriate treatment. Aliquots of these cultures were stored in 20% glycerol at -80° for later fitness assays. We tested all extant lines for respiratory deficiencies, *i.e.*, the petite phenotype, by plating on YPG (yeast extract peptone glycerol), a non-fermentable carbon source. Five lines, all from the normal-MA environment, failed to grow on YPG and were excluded from further study. Accounting for line extinctions, contaminants and respiratory deficiencies, we recovered 42 normal-MA and 47 salt-MA lines. Some previous yeast MA studies (*e.g.*, Joseph and Hall 2004) have made a founding diploid genotype completely homozygous by inducing a mating type switch in a haploid progenitor. We chose not to do this for two reasons. First, we believed that our initial isolate to be mostly homozygous given its history as a laboratory strain. Low heterozygosity in our founding genotype is consistent with the lack of detectable variation among haploids we derived from it (see Results). Second, and more importantly, any small amount of heterozygosity would be equally present in each of our experimental treatments, making comparisons controlled.

### Sporulation and haploid isolation

Following the completion of MA, we derived haploids from the frozen stocks of both MA treatments and from the ancestral population. We sporulated and successfully isolated haploids from 17 normal-MA and 21 salt-MA lines. Mating types were scored for all derived haploids. During haploid culture, one normal and three salt lines were contaminated, subsequently lost and were therefore scored for viability but not haploid growth rate (see below). Details of these procedures can be found in Supplemental Material File S1.

### Fitness component assays

#### Diploid maximal growth rate:

We quantified diploid asexual fitness by analyzing growth curves derived from optical density (OD) changes measured in a plate-reading spectrophotometer (Tecan Genios). MA line and ancestral replicates were arrayed in a series of randomized arrangements in 96-well plates designed to reduce environmental effects arising due to position within plates. OD was measured at regular intervals over a 48-hr period at 28° and used to construct population growth curves. Maximal growth rate was estimated by using a 7-hr sliding window along the growth curve (see Supplemental Material File S1). We attempted to measure growth 10 times for each extant MA line in addition to 200 total replicates for the ancestor. In total we generated 416, 366, and 200 individual growth curves from 42 normal-MA lines, 47 salt-MA lines and the ancestor, respectively.

#### Sporulation efficiency:

We quantified sporulation efficiency — a component of diploid sexual fitness — as the fraction of meiotic events (tetrads) under standard sporulation conditions in 96-well plates (see S1). Cells were examined under phase-contrast microscopy in a blind fashion with respect to the line identity (minimum 200 cells). To reduce environmental variability, we corrected MA sporulation estimates for density and individual plate effects based on ancestral genotype sporulation (see S1). In total we generated estimates of sporulation efficiency for 206, 196, and 99 replicate cultures from 42 normal-MA lines, 45 salt-MA lines and ancestor respectively.

#### Haploid viability:

Haploid viability was assessed from photographs of tetrad dissections by scoring the number of haploid lines recovered from each dissected tetrad. We obtained replicate viability measures for 89, 107, and 45 tetrads from 17 normal-MA lines, 21 salt-MA lines and ancestor respectively.

#### Haploid maximal growth rate:

Haploid maximal growth rate was measured by growth curve analysis, similar to the assessment of diploid maximal growth rate (S1). We generated 128 growth curves from haploids derived from the ancestral genotype, 350 growth curves from haploids derived from 16 normal-MA lines and 358 growth curves from haploids derived from 18 salt-MA lines.

### Changes in means and variance components following MA

The per-generation change in mean (Δ*M*), compared to the ancestor, was calculated for all four traits in the two MA treatments. To test for individual lines that differed in mean from the ancestor, we used the Steel method as implemented in JMP software (Version 11, SAS Institute Inc., Cary, NC, 1989-2019), which controls the overall error rate when making multiple comparisons. Mutational variance, *V*_m_, is the per-generation change in genetic variance and was estimated from the among-line variance in each MA treatment (Lynch and Walsh 1998). Mutations might render genotypes more susceptible to environmental perturbations, causing a decrease in canalization revealed by increased environmental variance (Hansen 2006). We therefore also measured the per-generation change in environmental variance (Δ*V*_E_/*t*) for each trait and MA treatment. We calculated mutational heritability, $h_{\text{m}}^{2}$, as the ratio of mutational variance to environmental variance (Houle *et al.* 1996), both expressed as differences from the ancestor. Note that for this ratio mutational variance is measured on a per-generation scale while environmental variance is not. For the above means, variances and mutational heritabilities, we tested for differences from the ancestor as well as between MA treatments using bootstrapping (programs written in Mathematica v. 11 Wolfram Research, Inc. 2016) with 10,000 or 20,000 iterations. Bootstrap resampling was conducted among lines for the two MA treatments. For the ancestor treatment, which lacks line structure, resampling was conducted so as to mimic the number of lines and observations per line of the comparator MA treatment.

For haploid growth rate we estimated a fuller set of variance components using REML and the MIXED procedure of SAS software:

$$\begin{array}{l} \text{Haploid Growth Rate}=\text{MA Treatment}+\text{Diploid Parent}\left(\text{MA Treatment}\right)+\text{Tetrad}\left(\text{Diploid Parent},\text{MA Treatment}\right)\\ +\text{Haploid Strain}\left(\text{Tetrad},\text{Diploid Parent},\text{MA Treatment}\right)+\text{residual}, \end{array}$$

where parentheses indicate nesting. We used likelihood-ratio tests to ask whether null models constraining the different variance components to zero differed from the unconstrained model. Parameters were constrained using the “parms” statement. Tests to determine whether the variance component differed from zero were one-tailed. We tested for differences between MA treatments in the estimated proportional variance components. For this we standardized the data for each treatment to a mean equal to zero and a standard deviation of one. In this case our null models were obtained by estimating only a single variance component between both MA treatments. We used likelihood ratios to compare the results to models that calculated separate variance components for each MA treatment. These tests were two-tailed. For tests of differences between MA treatments in maximal haploid growth rate, mutational variance and mutational heritability, we used the total non-residual variance component as our measure of genetic variance in our haploids. Bootstrap tests were performed by resampling 1,000 times among lines (diploid parents) with 1,000 replicates.

### Genetic correlations, M-matrix and evolvability

Genetic correlations (*r*_g_) among the four fitness components were estimated by REML using the “type = unr” covariance option in the MIXED procedure of SAS software (see Fry and Heinsohn 2002 for details). Likelihood-ratio tests were used to evaluate the hypothesis that the genetic correlation differed from zero and from one by constraining the estimate of the correlation using the “parms” statement. The tests for *r*_g_ equal to zero is two-tailed while the test for *r*_g_ equal to one is one-tailed. Differences in *r*_g_ between MA treatments were assessed with REML bootstrapping (2,000 iterations).

The M-matrix represents the per-generation change in genetic variances and covariances of traits due to mutation. Although the M-matrix is expected to shape the pattern of genetic variation, and thus determine accessibility of different phenotypic spaces, it has been estimated few times (McGuigan 2006). Using the mutational variances and correlations described above, we estimated the M-matrix for each MA treatment and compared matrices using the method of random skewers (Pielou 1984, Cheverud 1996, Cheverud and Marroig 2007). Among many matrix comparison methods, we chose random skewers because of their explicit relationship to evolutionary theory (Hansen and Houle 2008, Houle and Fierst 2012). Random skewers are vectors describing multivariate evolutionary response when random selection-gradient vectors are pre-multiplied by the M-matrix. To test for a difference between the M-matrices from the normal-MA and salt-MA experiments, we generated 100,000 random vectors drawn from a normal distribution with mean 0 and variance 1 and normalized them to unit length. Each such random vector was pre-multiplied by the two M-matrices, and the correlation between the two resulting random skewers was calculated. The random skewers metric was the mean of these 100,000 correlations. To test the null hypothesis of matrix dissimilarity, we obtained correlations between 100,000 pairs of random vectors, as above. If the observed random skewer metric exceeds 95% of the random correlations, then there is evidence for significant structural similarity (Cheverud and Marroig 2007). To explore whether matrix differences arose from differences in trait means, comparisons were conducted on both raw and mean-standardized matrices (see Hansen and Houle 2008). Finally, we calculated maximum evolvability, *e*_max_, as the first eigenvalue for both M-matrices (Schluter 1996, Hansen and Houle 2008).

### Dominance

Because we measured maximum growth rate in both diploids following MA and their derived haploids, we were able estimate dominance levels of deleterious mutations for this trait. If mutant effects are additive across loci, and diploid MA lines have a mean number, *n*, of unlinked mutant heterozygous loci, then the average fitnesses are ${\bar{w}}_{dip}=n\;a(1+k)$ for diploids and ${\bar{w}}_{hap}=n\;a$ for derived haploids, where *k* measures average dominance (h=(k+1)/2) and 2*a* represents the per-locus fitness of mutant haploids (1−s=2a). For haploids each mutant allele is thus considered to have the same fitness effect as a homozygous mutant in diploids. The average dominance measure *k* can therefore be estimated as $\left(\frac{{\bar{w}}_{dip}}{{\bar{w}}_{hap}}\right)-1$, so that $h={\bar{w}}_{dip}/2\;{\bar{w}}_{hap}$, which is independent of mutation number. Variances and regression can also be used to estimate dominance.

### Mutation rates and average effects

We obtained estimates of mutation parameters for all fitness traits using both the Bateman-Mukai method of moments (BM; Bateman 1959, Mukai 1964, Lynch and Walsh 1998) and maximum-likelihood (ML) method as implemented in mlgenomeu v.2.08 (Keightley 1994, Keightley and Ohnishi 1998). The BM method uses information on the changes in mean and among-line variance to estimate a lower-bounded value of the mutation rate and a maximum average effect. It assumes that all mutations are detrimental and of equal effect and is included here for comparison to earlier studies. Because we were interested in multiple potentially correlated traits, we did not correct for any selection that may have occurred during colony expansion between transfers as has been done in previous studies (*e.g.*, Kibota and Lynch 1996). Thus, we followed the example of Hall and Joseph (2010), who also used uncorrected values. Looking at multiple components of fitness where some are selected during colony growth and others are not, and allowing for correlation between traits, raises questions concerning how such corrections should be undertaken. For diploid growth rates and correlated traits of the same sign, uncorrected values should lead to an underestimate of the number of deleterious mutations and an overestimate of the number of beneficial mutations.

Keightley’s (1994) ML analysis assumes a Poisson-distributed number of mutations among lines and a gamma distribution of mutational effects with shape parameter *β* and scale parameter *α*. The program estimates the proportion of beneficial (positive-effect) mutations (*P*_B_) using the gamma distribution reflected about zero. The average effect of a mutation is E(*hs*) for heterozygous diploids and E(*s*) or E(*a*) for haploids and is estimated as the ratio of the gamma shape and scale parameters, *β*/*α*. Analyses were run on a cluster at the Atlantic Computational Excellence Network (ACEnet). We found ML parameter estimates by evaluating profile likelihoods in a manner similar to Joseph and Hall (2004) by holding the gamma distribution shape parameter (*β*) and the proportion of positive-effect mutations (*P*_B_) constant for each run and allowing the program to find the ML values of *α* and *U*. We ran most combinations of *β* = 0.01, 0.1, 0.5, 1, 2, 5, 10, 15, 20, 40 and *P*_B_ = 0, 0.01, 0.05, 0.1, 0.15, 0.2, 0.25, 0.3, 0.35, 0.4, 0.45, 0.5 and 0.55. We also explored an equal-effects model for all values of *P*_B_. We then used increasingly finer steps of *β* and *P*_B_ values to further evaluate the likelihood surface. Following Keightley and Ohnishi (1998), we used line means as the observed trait value to reduce computation time. The drawback of this approach is that it assumes the error variance of the trait values is equal to the error variance of the ancestral replicates. Confidence intervals for the estimates were obtained by examining profile likelihood curves and taking the parameter values at 2 log-likelihood values in each direction from the maximum as the lower and upper bounds of the interval (Keightley and Ohnishi 1998).

We calculated BM estimates using two methods. Method A utilized the variance component attributed only to differences among diploid parental lines. Method B utilized the total variance minus the within-haploid-line (*i.e.*, residual) variance as calculated in our nested model (see above). Method B therefore captured variance attributed to differences among parents, tetrads within parents and haploid strains within tetrads.

### Data availability

File S1contains detailed methods for 1) estimation of cell number and number of generations from colony size during mutation accumulation; 2) sporulation, haploid isolation and scoring mating types; and 3) fitness assays for diploid and haploid growth-curve analysis, including corrections for environmental (position) effects on growth rate and sporulation rate. File S2 contains all individual fitness values (raw minus ancestral mean) for the four traits. Table S1 contains values for change in mean, mutational variance, change in environmental variance and mutational heritability for all fitness components, as well as tests for differences between MA treatment. Supplemental material available at figshare: https://doi.org/10.25387/g3.12698096.

## Results

### Means, variances and variance components

**Figure 1:**
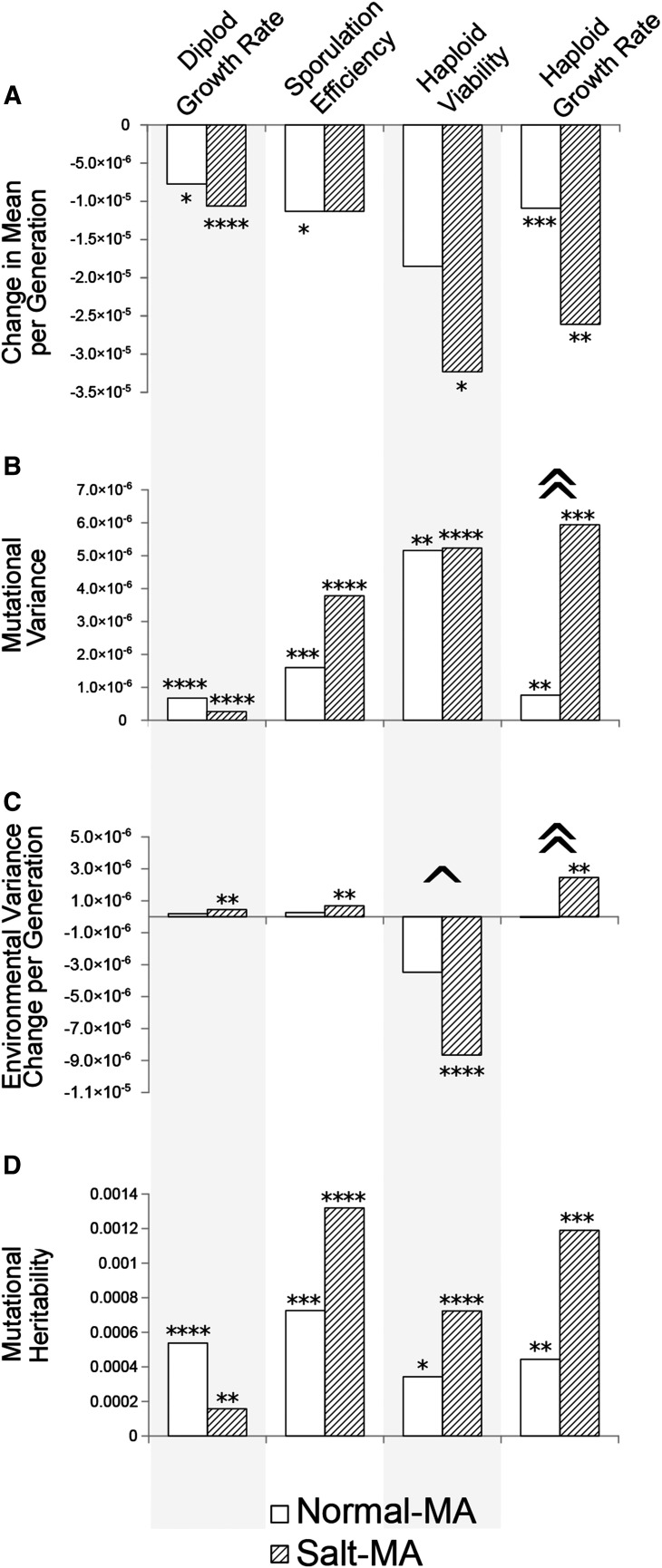
Change in mean, mutational variance, change in environmental variance and mutational heritability for four fitness components under normal and stressful mutation accumulation. Changes in mean (Δ*M*), genetic variance (*V*_m_) and environmental variance (Δ*V*_E_) are expressed per generation. Asterisks indicate statistically significant changes from the ancestor (* *P* < 0.05, ** *P* < 0.01, ***P < 0.001, **** *P* < 0.0001), and carets indicate significant differences between normal- and salt-MA (^ *P* < 0.05, ^^ *P* < 0.01). For presentation clarity, haploid viability values are divided by 10 for Δ*M* and by 100 for *V*_m_, and Δ*V*_E_. Sample sizes, number of lines differing in mean from ancestor and values for measure are presented in Supplemental Material Table S1.

**Figure 2:**
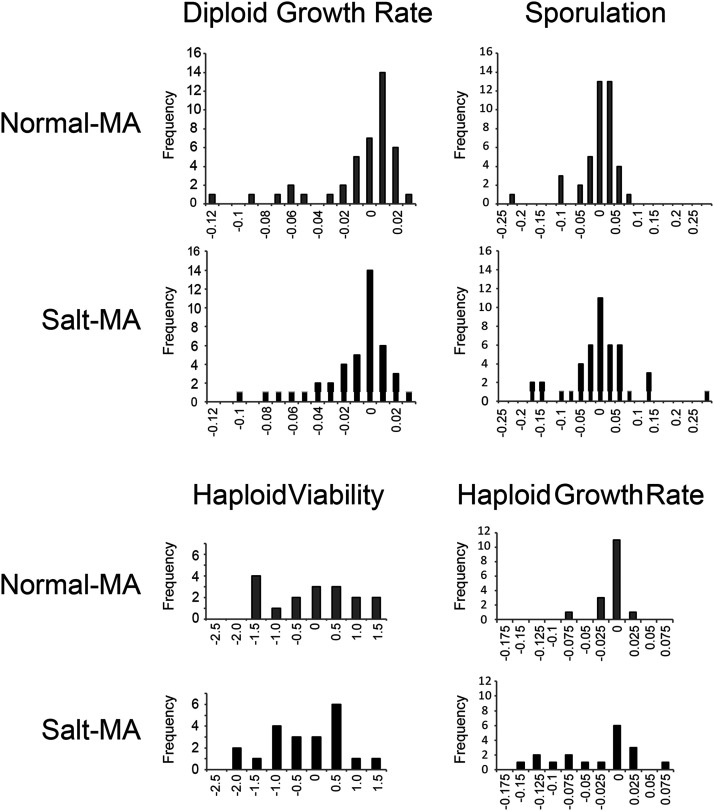
Distribution of MA line means for fitness traits in yeast when mutations accumulated under standard (normal-MA) or high-salt (salt-MA) conditions. Fitness components were standardized by subtracting the average ancestral value.

Mean values declined for all four fitness components in both MA treatments. These per-generation declines were statistically significant in all cases except sporulation under salt-MA and haploid viability under normal-MA. Changes in mean did not differ between treatments (Figure 1A, Table S1). For diploid maximal growth rate, five individual lines in normal-MA and three in salt-MA grew more slowly than the ancestor, and no MA lines from either treatment had faster diploid growth than the ancestor (Figure 2, Table S1). For sporulation, four lines in the normal-MA had lower means than the ancestor, while none was higher. Six salt-MA lines had lower sporulation than the ancestor and two sporulated at a higher efficiency (Figure 2, Table S1). Our ancestral strain had an uncorrected average sporulation efficiency of 23.4% under our conditions. Mean haploid viability was 2.3 spores per tetrad among ancestral replicates, 2.1 for normal-MA and 1.8 for salt-MA. No individual MA lines differed from the ancestor for this trait. For haploid maximal growth rate, three normal lines had slower growth than the ancestor and none was higher, while four salt-MA lines grew more slowly than the ancestor and one showed improved growth (Figure 2; Table S1).

Mutational variance was estimated for the two diploid traits and haploid viability using the among-line variance component and for haploid growth rate using the sum of the nested genetic components diploid MA line, tetrad within line and haploid strain within tetrad under REML. All fitness components in both MA treatments exhibited statistically significant mutational variance (*V*_m_, Figure 1B, Table S1). This per-generation increase in genetic variance was numerically greater in three of four traits and was 7.8 times larger under salt-MA than normal-MA for haploid growth rate (*P* < 0.004; Figure 1B, Table S1).

Environmental variance changed significantly in all four traits under salt-MA, increasing for diploid growth rate, sporulation efficiency and haploid growth rate but decreasing for haploid viability. The change in environmental variance differed between normal- and salt-MA for haploid viability (*P* = 0.023) and haploid growth rate (*P* = 0.006; Figure 1C, Table S1). Mutational heritability, $h_{\text{m}}^{2}$, was detected for all traits in both MA treatments (Figure 1D, Table S1). Values for salt-MA were larger than for normal-MA for three of four traits but did not differ significantly between treatment for any trait.

**Figure 3:**
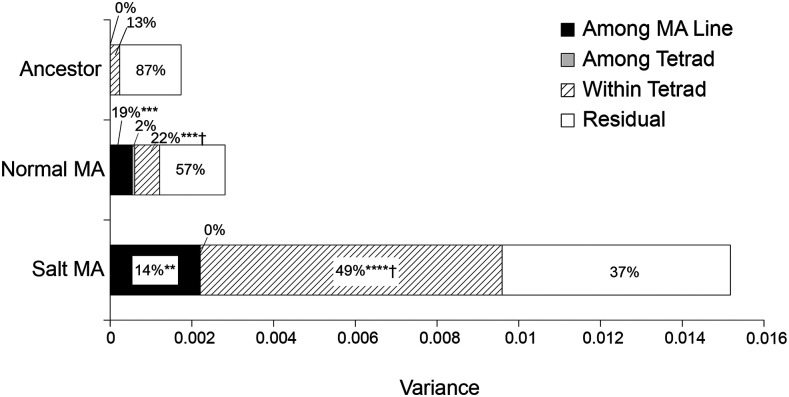
Components of variance for haploid growth rate in yeast that accumulated mutations under salt stress (salt-MA) or standard conditions (normal-MA). Significance levels for components differing from zero are * *P* < 0.05, ** *P* < 0.01, ***P < 0.001, **** *P* < 0.0001. Variance components proportions differing between MA treatments (*P* < 0.05) are indicated by †.

For haploid growth rate, we further divided the genetic component of variance into components due to diploid MA line, tetrad within line and haploid strain within tetrad. These variance components are shown in Figure 3, where the values are accumulated totals following MA. For the ancestor, there was no variance among pseudo-lines or among tetrads within lines; instead all variance in haploid growth rate occurred within tetrads (13%) or as environmental variance (87%; Figure 3). We detected significant variance among diploid MA lines for haploid growth rate in both MA treatments (likelihood-ratio tests: normal-MA, *P* = 0.0004; salt-MA, *P* = 0.009; Figure 3). No significant difference was found among tetrads in either MA treatment (*P* = 1; Figure 3), but the variance among haploid strains within a tetrad was significant in both MA treatments (normal-MA, *P* = 0.0002; salt-MA, *P* < 0.0001; Figure 3). This within-tetrad variance was significantly higher (49%) in salt-MA than normal-MA (22%) as a proportion of the total variance (likelihood-ratio test: *P* = 0.01).

### Genetic correlations, M-matrix and evolvability

**Figure 4:**
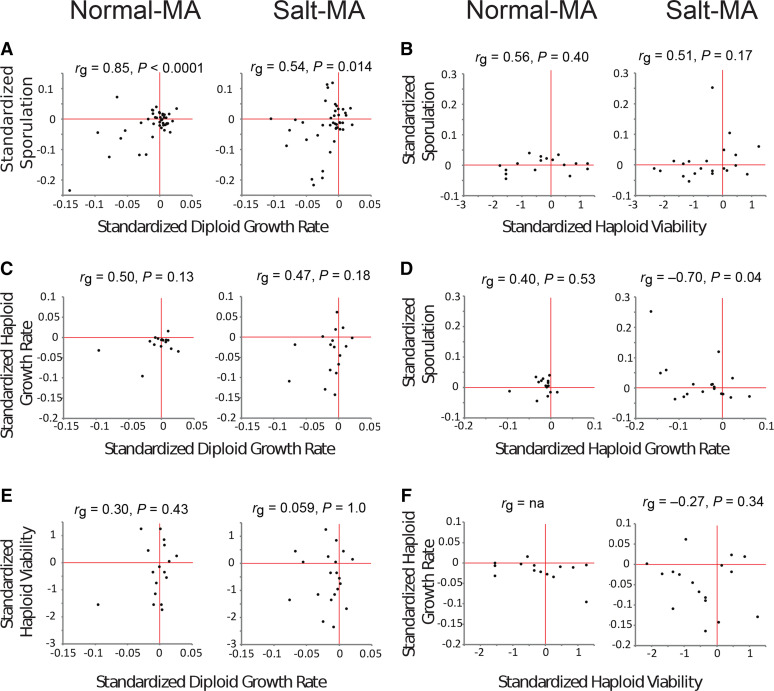
Genetic correlations among the four fitness traits. Genetic correlations were calculated under a mixed-effects maximum-likelihood model, and significance levels were determined with likelihood-ratio tests. The correlation between haploid viability and haploid growth rate for normal-MA was indeterminable due to infinite likelihood.

In diploids the genetic correlation between growth rate and sporulation efficiency was significantly positive in both the normal-MA (*r*_g_ = 0.85, *P* < 0.0001) and salt-MA (*r*_g_ = 0.54, *P* = 0.0028) treatments (Figure 4). These correlations did not differ between MA treatments (*P* = 0.18). The genetic correlation between sporulation rate and haploid growth rate was significantly negative in the salt-MA treatment (*r*_g_ = −0.70, *P* = 0.0005) but was non-significantly positive in normal-MA (*r*_g_ = 0.40, *P* = 0.48). Most of the remaining correlations were moderately strong (0.3 to 0.56) but were not statistically different from zero (*P* > 0.05; Figure 4).

Variances and covariances among traits arising from mutation are shown in the M-matrix (Table 1). The genetic correlation between haploid viability and haploid growth rate was not estimable; we therefore compared M-matrices comprising the three traits diploid growth rate, sporulation and haploid growth rate. Random skewers indicated that normal- and salt-MA matrices differed in structure (random skewers metric 0.53, *P* = 0.32 for null hypothesis of no similarity). Maximum evolvability as measured by the dominant eigenvalue was 2.35 × 10^−6^ under normal-MA and 8.36 × 10^−6^ under salt-MA. Mean-standardized M-matrices also showed evidence of differences between treatments. In this case the random skewer metric was 0.60 (*P* = 0.30 for null hypothesis of no similarity), and evolvabilities were 0.011 and 0.014 for normal- and salt-MA, respectively.

**Table 1:** Mutation matrix for four traits under normal-MA (above diagonal) and salt-MA (below diagonal)

Trait	(1) Diploid growth rate	(2) Sporulation efficiency	(3) Haploid viability	(4) Haploid growth rate
(1) Diploid growth rate	6.72	8.83	55.01	3.58
	2.63			
(2) Sporulation efficiency		16.10	162.01	4.45
	5.41	37.90		
(3) Haploid viability			5160.00	.
	6.86	225.19	5230.00	
(4) Haploid growth rate				7.62
	5.94	−33.02	−152.89	59.40

Entries are per-generation changes in variance and covariance due to mutation and are multiplied by 10^7^.

### Mutation rates, effect sizes and dominance

Mutations affecting diploid maximal growth rate arose at approximately 2.4 times the rate under salt stress (*U* = 0.00036 mutations/genome/generation; 0.00029 – 0.04 95% confidence interval) compared to normal-MA conditions (0.00015, 0.00011 – 0.00039 95% C.I.; Table 2). The average heterozygous effect *E*(*hs*), conversely, was approximately halved, from 0.060 (0.024 – ∞ 95% C.I.) in normal-MA to 0.029 (0.00027 – 0.035 95% C.I.) under salt-MA. Our ML estimate of the gamma distribution shape parameter *β* was lower for salt-MA lines, indicating more mutations of smaller effect within this treatment. The estimated proportion of positive-effect mutations (*P*_B_) affecting growth rate was zero in both MA environments (Table 2).

**Table 2:** Maximum-likelihood and Bateman-Mukai estimates of mutation parameters for *S. cerevisiae* diploids and their derived haploids after approximately 1,500 generations of mutation accumulation under normal or salt-stress conditions

Fitness Trait	Mutation Parameter	Maximum likelihood		Bateman-Mukai	
		Normal	Salt stress	Normal	Salt stress
Diploid growth rate	*U* × 10^4^	1.5	3.6	0.92	3.1
		(1.1 – 3.9)	(2.9 – 400)		
	*E*(*hs*) or *s*_max_	0.060	0.029	0.085	0.035
		(0.024 – ∞)	(0.00027 – 0.035)		
	Shape (*β*)	4.4	1.8		
		(0.3 – ∞)	(≤0.005 – 40)		
	*P*_B_	0	0		
		(0 – 0.28)	(0 – 0.23)		
Sporulation	*U* × 10^4^	4.4	610	0.82	0.32
		(1.1 – 610)	(5.9 – 1300)		
	*E*(*hs*) or *s*_max_	0.040	0.00088	0.14	0.37
		(0.00038 – 0.10)	(0.00043 – 0.074)		
	Shape (*β*)	0.8	0.01		
		(≤0.005 – 35)	(≤0.005 – 3.5)		
	*P*_B_	0.21	0.39		
		(0 – 0.51)	(0.25 – 0.52)		
Haploid viability	*U* × 10^4^	117	280	1.0	3.8
		(3.5 – 3600)	(3.8 – 2200)		
	*E*(*hs*) or *E*(*a*)	∞	0.12	1.8	0.90
		(0.0052 – ∞)	(0.007 – 1.03)		
	Shape (*β*)	→ ∞	60		
		(0.01 – ∞)	(0.01 – 80)		
	*P*_B_	0.45	0.45		
		(0 – 0.55)	(0 – 0.5)		
Haploid growth rate	*U* × 10^4^	0.4	5.1	(Method A) 3.3	3.4
				(Method B) 1.8	1.1
		(0.4 – 290)	(4.4 – 3600)		
	*E*(*a*) or *s*_max_	→∞	0.069	(Method A) 0.033	0.081
				(Method B) 0.06	0.23
		(0.00033 – ∞)	(0.00027 – ∞)		
	Shape (*β*)	∞	43		
		(≤0.01 – ∞)	(≤0.01 – ∞)		
	*P*_B_	0	0.14		
		(0 – 0.55)	(0.01–0.45)		

*U*, genomic mutation rate expressed as 10^4^ × estimated value; *P*_B_, estimated proportion of beneficial mutations; *E*(*hs*) and *E*(*a*) average effect of a mutation in diploids and haploids, respectively. Confidence intervals (95%) for maximum-likelihood estimates are in parentheses.

Sporulation mutation rate estimates were 139 times higher for salt-MA (*U* = 0.061, 0.00059 – 0.13) than for normal-MA (0.00044, 0.00011 – 0.061; Table 2). Compared to mutations affecting growth rate, sporulation mutation rate estimates were three times higher for normal-MA and at least two orders of magnitude higher for salt-MA, and effect sizes were smaller (Table 2). Estimates of the shape parameter *β* were also much smaller for sporulation, indicating a leptokurtic distribution of effects on sporulation, particularly for salt-MA. In contrast to the lack of positive-effect mutations for growth rate, this proportion (*P*_B_) was 20–40% in the two MA treatments. These estimates may not represent the true ML values, however, as the likelihood surface was very flat near the maximum value found.

Mutations affecting haploid viability arose at approximately 2.4 times the rate under salt stress (*U* = 0.0280, 0.00038 – 0.22) compared to normal-MA conditions (0.0117, 0.00035 – 0.36; Table 2). The proportion of mutations positively affecting viability was 0.45 (0 – 0.55 normal-MA; 0 – 0.5 salt-MA) in both MA treatments. For haploid maximal growth rate, salt stress increased the ML estimated mutation rate by an order of magnitude (*U* = 0.00051 salt-MA (0.00044 – 0.36) *vs.* 0.000040 normal-MA (0.00004 – 0.029) and increased the proportion of positive-effect mutations from zero (0 – 0.55) in normal-MA to 0.15 (0.01 – 0.45) in the salt treatment (Table 2).

Dominance levels of mutations affecting growth rate were 0.35 for normal-MA and 0.20 for salt-MA.

## Discussion

Using budding yeast, we performed a 1,500-generation MA experiment in both stressful and benign environments and estimated mutational parameters for four fitness traits. Most MA studies to date have employed only benign environments. Natural environments are variable and probably often far from benign, and we wished to explore mutational properties when an organism lives away from its optimal fitness. A few organisms have been studied under more challenging conditions, including flies (Keightley and Ohnishi 1998), worms (Davies *et al.* 1999, Matsuba *et al.* 2012, Katju *et al.* 2018), plants (MacKenzie *et al.* 2005) and yeast (Liu and Zhang 2019). Our use of NaCl was an attempt to address the role of environmental stress with an agent commonly encountered by yeast but at a level that significantly impacts growth. The extensive cellular apparatus used to modulate NaCl tolerance in *S. cerevisiae* suggests the common occurrence of this salt in yeast environments.

We detected statistically significant mutational variance and mutational heritability for all four fitness components in both MA treatments. Three components showed greater mutational variance under stress, significantly so for haploid growth rate, which increased by more than sevenfold. This increase was not detected in diploid growth rate, consistent with masking of mutations in the heterozygous state. The genetic architecture arising from mutation (M-matrix) also differed between normal and salt conditions, as tested by random skewers. Theoretical studies suggest that the M-matrix can shape the G-matrix (McGuigan 2006). If so, stress will influence evolutionary trajectories by altering both selection and the genetic variance-covariance structure of traits. To conclude that stress increases mutational variance as a general principle, both sets of MA lines would ideally be tested in a range of environments. This approach would control for any genotype-by-environment interactions affecting mutational variance.

In addition to changes in variance and genetic architecture, we found that for every fitness component examined, salt stress increased the phenotypically estimated mutation rate and decreased the average effect size compared to a benign environment. Although the consistently higher point estimate of the mutation rate under stress agreed with predictions, this parameter did not differ statistically between environmental treatments for any trait. Wide confidence intervals for mutation parameters are unfortunately typical of MA experiments (*e.g.*, Keightley and Ohnishi 1998, Schultz *et al.* 1999, Hall and Joseph 2010). Furthermore, average effect size is negatively correlated with mutation rate in the maximum-likelihood analyses, so these parameters cannot be compared independently. Using *S. cerevisiae*, Liu and Zhang (2019) recently performed an MA study in several environments considered mildly stressful, and then used whole-genome sequencing to determine the rate and spectrum of mutations. One of their environments matched ours — 1M NaCl in YPD medium — which in both studies initially reduced growth rate by one-half. As in our study, Liu and Zhang (2019) found that this stress doubled the mutation rate. This agreement between direct sequencing and fitness-based maximum likelihood is especially striking because the latter quantifies only mutations with some fitness effect.

Our measures of growth rate in both diploids and their derived haploids allowed us to estimate dominance levels of mutations affecting this trait. The estimates of 0.35 for normal-MA and 0.20 for salt-MA are typical of data from a variety of species which indicate that most deleterious mutations are partly recessive (Simmons and Crow 1977, Charlesworth 1979, Szafraniec *et al.* 2003). Any loss of haploids because of exposed mutations will cause an underestimate of dominance level, because mean haploid fitness is increased, and this appears in the denominator. This method of dominance estimation assumes that the same genes affect growth rate in both ploidy levels. Several lines of evidence militate against this view. First, the genetic correlation arising from mutation between diploid and haploid growth rate was approximately 0.5 for both MA treatments and did not differ statistically from zero. If these were the same trait, then diploids and their derived haploids should show a genetic correlation of approximately one. Second, maximum-likelihood estimates of mutation parameters indicated that salt-MA induced more mutations of smaller effect compared to normal-MA. If this is true, the salt-MA dominance levels should have been higher because of the inverse relation between deleterious effect size and dominance (Simmons and Crow 1977). Finally, we found evidence for a small proportion of positive-effect mutations in haploid growth rate under salt stress, and beneficials can disrupt dominance estimates (Joseph and Hall 2004).

### Derived haploids

We were able to combine variance component analysis with the power of the yeast genetic system to dissect the sources of heritable variation more finely than in earlier studies, which typically partitioned variance into mutational (among-line) *vs.* environmental (within-line) components. Specifically, by tracking the products of individual meiotic events, we decomposed the variance in haploid growth rate due to MA line, tetrad within line, haploid spore within tetrad and environment (residual within strain). The proportion of variance in growth rate arising from spores within tetrads more than doubled for salt-MA (49%) compared to normal-MA (22%). For any heterozygous locus, mutant and wild-type alleles will segregate among spores, elevating this variance component. Therefore, this variance-component difference between MA environments strongly suggests a role for segregating new mutations. While environmentally induced epigenetic effects cannot be ruled out in MA studies, these factors are generally believed to reset during meiosis (Harvey *et al.* 2018).

### Mutations and canalization

**Figure 5:**
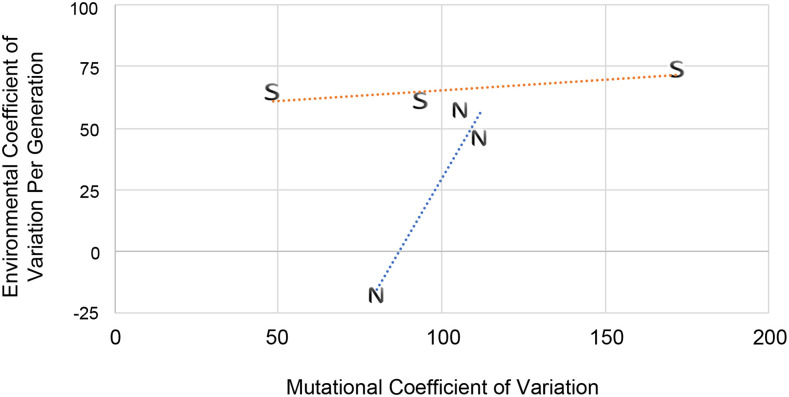
Decanalization of phenotypes by mutation. Values are mutational and environmental coefficients of variation (*CV*) calculated using the per-generation change in variance and in mean compared to the ancestor. Separate linear regression lines are shown for each MA treatment. N, normal-MA; S, salt-MA.

Mutations might render genotypes more susceptible to environmental perturbations, causing a decrease in canalization revealed by increased environmental variance. To test for a change in canalization with mutation, we investigated the relation between mutational and environmental variance (Hansen 2006, Baer 2008). Both kinds of variance were expressed as difference from the ancestor per generation and were converted to coefficients of variation (*CV*) to reduce effects of trait means on variances. For these *CV*s we standardized by the change in mean per generation rather than simply the mean at the end of MA. This puts both the variance and mean changes on a per-generation scale and also controls for the small difference in number of MA generations between treatments. For haploid viability, the environmental variance declined with MA in both treatments, significantly so for salt-MA. This trait thus did not match predictions of reduced canalization. For the other three traits, mutational and environmental *CV*s showed trends of increasing with one another in both treatments (Figure 5). The *R*-square values for normal-MA (0.90) and salt-MA (0.64) suggest a relationship between environmental and mutational variance, but the small number of traits precludes drawing firmer conclusions. Evidence for an effect of mutation on *V*_E_ has come from bristle number (Frankham 1980, Mackay 1985; see Zhang and Hill 2008) and gene expression (Rifkin *et al.* 2005) in *Drosophila* as well as productivity and body size in rhabditid nematodes (Baer 2008).

A correlation between environmental and mutational variance also has implications for mutational heritability as measure of the amount of variation introduced by mutation. Specifically, changes in mutational variance will not be fully reflected in mutational heritability because the latter is scaled by environmental variance (Houle *et al.* 1996). Haploid growth rate is particularly interesting here, as mutational variance was ∼7.8 times greater under salt-MA but mutational heritability increased by a factor of only ∼2.7. Joseph and Hall (2004) report a mutational heritability of 1.1 X 10^−3^ for maximum growth rate in diploid yeast, a value identical to ours for salt-MA but higher than for normal-MA (4.4 × 10^−4^). Our values of mutational heritability are typical of those found in a variety of organisms, that is in the range of 10^−3^ to 10^−4^ (Houle *et al.* 1996). It is important to note that some measures of mutational heritability scale by environmental (residual) variance rather than by change in environmental variance compared to the ancestor, as done here.

### The role of selection in our experiment

The MA procedure should result in evolved lines where deleterious mutations are more frequent and beneficial mutations underrepresented relative to a non-MA condition. Nevertheless, some selection remains, most prominently for dominant lethals and near-lethals. We minimized the opportunity for selection on diploid growth rate by using a dissecting microscope to randomly pick colonies so that colonies of all sizes were equally likely to initiate the next generation. The important question is whether selection affected our normal and stressful MA treatments differently and whether differential selection could explain observed differences between the two MA treatments. We can use the incidence of respiratory deficiencies, *i.e.*, petite mutations, to gain some insight regarding selection differences because of the involvement of mitochondria in the response to salt stress (Pastor *et al.* 2009, Gao *et al.* 2011, Gao *et al.* 2014, Lahtvee *et al.* 2016). Petites are commonly detected in yeast MA experiments (Zeyl and DeVisser 2001, Hill and Otto 2007, Dickinson 2008) under salubrious conditions. In our study, no petites were recovered among the 47 salt-MA lines while five of 47 normal-MA lines were petites (Fisher exact test *P* = 0.056). This difference between treatments is consistent with petite removal by selection under salt stress. Given that the vast majority of mutations are deleterious, stronger selection in the stressful treatment would cause a reduction in several quantities, including Δ*M*, *V*_m_, *h*^2^_m_, *U* and *E*(*hs*). Furthermore, if mutations cause a reduction in canalization, then Δ*V*_E_ will also be underestimated. Therefore, to the extent that differential selection on petite mutations represents mutations generally, our study underestimates the differences between MA treatments and the effects of stress on the mutational process.

### Sporulation genes and pleiotropy

Fitness components commonly show positive mutational correlations (Charlesworth 2015). In MA studies genetic correlations among traits are often interpreted as evidence for pleiotropic effects of new mutations. Positive correlations, however, may also arise from “general pleiotropy” where independent mutations affect phenotypes in similar ways (Estes and Phillips 2006). We found a significant correlation between sporulation and both diploid and haploid growth rate. Diploid growth rate was correlated with sporulation in both MA treatments, indicating positive pleiotropy or correlated effects. Genetic associations between asexual and sexual function have been studied in a variety of fungal systems (Saleh *et al.* 2012), and there is evidence for both positive (Zeyl *et al.* 2005, Hill and Otto 2007, Hall and Joseph 2010) and negative relationships (Xu 2002, Zeyl *et al.* 2005).

We also detected a negative correlation between haploid growth rate and sporulation, but only in our salt-MA treatment. This implies that at least some mutations can increase sporulation while decreasing haploid growth. When the most-highly sporulating line was removed from this analysis, the correlation remained negative but was no longer statistically significant. A negative correlation between haploid fitness and sexual function in yeast was also found by Zeyl *et al.* (2005), although the lines they studied differed from ours in several important ways, making direct comparisons difficult.

### Stress-induced mutation as a general phenomenon

The hypothesis that environmental quality can influence the production of this variation is nearly as old as the study of mutation itself (*e.g.*, Muller 1928). Studies in both eukaryotes and prokaryotes indicate that generalizations regarding rates and effects can be difficult to make because they depend specifically on the kinds of organisms and their particular stresses (*e.g.*, Maharjan and Ferenci 2017). That this is so should not be surprising, as adaptations to reduce harm to successful genotypes are probably as old as life itself. The findings of general increases in genome-wide mutation rates in response to stress have been addressed only in laboratory experiments. The degree to which this phenomenon affects organisms in natural environments remains an open question, but it seems reasonable that stress-induced mutation may be general. The recent results of Liu and Zhang (2019) are especially relevant in this regard, as they show that mutation rates change with small environmental stresses, with greater stresses (reduced growth) associated with higher nuclear mutation rates. Mutation rates might also differ between haploid and diploid ploidy levels (Sharp *et al.* 2018), which may be differentially affected by environment. Stressful environments are interesting not only because of their potential effect on the mutational process but also because they change the selective environment in which new mutations occur. Because many organisms face numerous and pervasive environmental stresses, the role of the environment in modulating both the input and fate of new heritable variation should be more thoroughly investigated.
